# Environmental barriers, person-environment fit and mortality among community-dwelling very old people

**DOI:** 10.1186/1471-2458-13-783

**Published:** 2013-08-28

**Authors:** Merja Rantakokko, Timo Törmäkangas, Taina Rantanen, Maria Haak, Susanne Iwarsson

**Affiliations:** 1 Gerontology Research Center and Department of Health Sciences, University of Jyväskylä, Jyväskylä, Finland; 2 Department of Health Sciences, Faculty of Medicine, Lund University, Lund, Sweden

**Keywords:** ENABLE AGE-project, Environment, Mortality, Older people, Housing Enabler

## Abstract

**Background:**

Environmental barriers are associated with disability-related outcomes in older people but little is known of the effect of environmental barriers on mortality. The aim of this study was to examine whether objectively measured barriers in the outdoor, entrance and indoor environments are associated with mortality among community-dwelling 80- to 89-year-old single-living people.

**Methods:**

This longitudinal study is based on a sample of 397 people who were single-living in ordinary housing in Sweden. Participants were interviewed during 2002–2003, and 393 were followed up for mortality until May 15, 2012.

Environmental barriers and functional limitations were assessed with the Housing Enabler instrument, which is intended for objective assessments of Person-Environment (P-E) fit problems in housing and the immediate outdoor environment. Mortality data were gathered from the public national register. Cox regression models were used for the analyses.

**Results:**

A total of 264 (67%) participants died during follow-up. Functional limitations increased mortality risk. Among the specific environmental barriers that generate the most P-E fit problems, lack of handrails in stairs at entrances was associated with the highest mortality risk (adjusted RR 1.55, 95% CI 1.14-2.10), whereas the total number of environmental barriers at entrances and outdoors was not associated with mortality. A higher number of environmental barriers indoors showed a slight protective effect against mortality even after adjustment for functional limitations (RR 0.98, 95% CI 0.96-1.00).

**Conclusion:**

Specific environmental problems may increase mortality risk among very-old single-living people. However, the association may be confounded by individuals’ health status which is difficult to fully control for. Further studies are called for.

## Background

Environmental barriers and Person-Environment (P-E) fit problems in housing are associated with disability-related outcomes in older people (for a review, see [1]), and may also lead to increase in nursing home care [2]. It has also been found that barriers in the outdoor environment predict mobility decline [3-5], which has been identified as a significant predictor of mortality [6,7]. However, little is known about the effect of environmental barriers on mortality, the ultimate endpoint of health decline.

Among the very few investigations on housing environments and mortality, a recent Spanish study [8] found that self-reported poor housing conditions are associated with higher mortality among older people with heart failure. In particular, reporting lack of an elevator in an apartment building and frequently feeling cold in the house increased mortality risk. In addition, a study of senior residents in Tokyo showed that self-reported positive environmental characteristics in the neighborhood, such as walkable streets and green spaces near one’s residence, were associated with survival [9]. No research, however, has been done on the associations between mortality and objectively measured environmental barriers indoors, in entrances and in the immediate outdoor environment of home among older people.

Correlations have been reported between environmental features and outdoor activities, which are considered important for well-being in old age (for a review, see [10]). Environmental barriers may restrict possibilities to access and enjoy the outdoors and lead to inactivity [11]. Low frequency of going outdoors is associated with depressive mood, poor subjective health and low cognition [11-13], and it also increases mortality risk [14,15].

According to the ecological theory of ageing [16], P-E fit reflects the relationship between the individual’s functional capacity and the demands of the environment [16-18]. Environmental barriers are commonly seen as negative features of the environment that cause problems for persons with functional limitations [19]. However, it should not be taken for granted that challenging environmental features are invariably detrimental to health. Although little studied to date it might reasonably be argued that in some cases such challenges may induce a training effect and help maintain or even improve functioning. For example, stairs may eventually become barriers to mobility, despite the facts that for many people climbing stairs provides physical exercise that helps maintain functional capacity [20]. As functional limitations become more severe, the capacity to adapt may decrease and environmental challenges may become overwhelming [16], potentially leading to avoidance of challenging situations, restricted activity and further decline in health. For older people with functional limitations, lowering environmental press may increase independence in daily activities and wellbeing [17]. It is well known that single-living very old people are especially sensitive to environmental press, and that those living alone have a pronounced risk of losing their independence and becoming socially isolated. Thus, they constitute an important target for research on aspects of home and health during the ageing process [21].

The aim of this study was to examine whether objectively assessed environmental barriers outdoors, at entrances and indoors and environmental barriers that generate P-E fit problems predict mortality among single-living community-dwelling very old people.

## Methods

### Project context and participants

The present study is based on the Swedish data of the European project “Enabling Autonomy, Participation, and Well-Being in Old Age: The Home Environment as a Determinant for Healthy Ageing” (ENABLE-AGE). The aim of the ENABLE-AGE project was to examine the home environment and its importance for healthy ageing. The study design and methods have been described in detail elsewhere [22].

The target population consisted of community-residing, very old, single-living inhabitants of three mainly urban municipalities in southern Sweden (Halmstad, Helsingborg, Lund). The sample was drawn from the public national register: 1,593 persons were contacted by telephone to verify that they fulfilled all the inclusion criteria. To be eligible for the study, participants had to live alone in ordinary housing, be 80-89-years old and willing to participate. After screening, 965 were considered eligible for the study and, of whom 41% (n = 397) agreed to participate. The main reasons for non-participation were lack of interest or time, poor health, distrust/fear or considering the data collection too strenuous.

A total of 397 people, aged 80–89 at baseline, were interviewed four times over a nine-year period, specifically in 2002/2003, 2003/2004, 2009 and 2011. The 393 participants who provided their social security numbers at baseline were followed up for mortality until May 15, 2012. The data were collected by occupational therapists who had been trained in how to conduct the interviews and observations, both of which were to be performed in the participants’ homes [23].

The ENABLE-AGE project was approved by the ethical committee of Lund University, Sweden. The study was conducted according to the guidelines for good scientific and clinical practice laid down by the Declaration of Helsinki. Participants were informed about the study and a signed informed consent was obtained.

### Measurements

#### ***Mortality***

The study population was followed up for mortality from the baseline interview (2002/2003) to May 15, 2012. Information on date of death was obtained from the Swedish public national register. Follow-up time was calculated as days from the baseline interview until date of death or end of follow-up, whichever happened first.

#### ***Environmental barriers***

Environmental barriers were assessed by means of the Housing Enabler (HE) instrument [24]. The HE instrument includes detailed observation and assessment of the presence or absence of 188 environmental barriers (items) in the home and immediate outdoor environment. The housing environment is divided into four sections: outdoor environment (33 items), entrances (49 items), indoor environment (100 items), and communication (6 items; not used because of valid internal drop-out due to different housing type characteristics). Sum scores for the environmental barriers were calculated separately for the outdoor (range 0–33), entrance (0–49) and indoor (0–100) environments.

To identify the single environmental barriers that generate the most P-E fit problems, a so-called weighted environmental barrier analysis was conducted [19]. This analysis is part of the HE software [24], and is based on the sample specific prevalence of functional limitations in relation to the occurrence of environmental barriers. This computation generates environmental barrier item-specific P-E fit scores and results in a list ranking the environmental barriers from those generating the most P-E fit to the least. This ranking is based on the relative contribution of each environmental barrier item to the variance in the total HE score. The five environmental barriers that generated the most P-E fit problems in the study sample, in each housing section, were used in the analysis.

#### ***Confounders***

Functional limitations (12 items) and use of mobility devices (two items) were dichotomously assessed (present/not present) based on interview and observation, according to the HE instrument manual [25]. For the variable ‘number of functional limitations’, a sum score ranging from 0 to 12 was calculated. The list of functional limitations is shown in Table 1.

**Table 1:** **Baseline characteristics of the Swedish ENABLE-AGE sample (n = 397**^**a**^**)**

**Characteristics**	**%**	**n**	
Women	75	296	
Type of housing			
Multi-dwelling block	83.1	330	
One-family house	14.4	57	
Semidetached/two family house	2.5	10	
Type of area			
Urban	83.4	331	
Semi-urban	13.6	54	
Rural	3.0	12	
Reliance on walking aid	41.3	164	
Functional limitations			
Difficulty in interpreting information	2.5	10	
Severe loss of sight	15.1	60	
Complete loss of sight	2.3	9	
Severe loss of hearing	20.7	82	
Prevalence of poor balance	42.3	168	
Incoordination	3.8	15	
Limitations of stamina	42.1	167	
Difficulty in moving head	14.4	57	
Difficulty in reaching with arms	28.0	111	
Difficulty in handling and fingering	22.9	91	
Loss of upper extremity skills	3.8	15	
Difficulty in bending and kneeling	64.2	255	
	**Mean**	**SD**	**Range**
Number of functional limitations	1.7	1.4	0-5
MMSE, four item sum	3.4	0.8	0-4
GDS, score	3.0	2.3	0-13
Age, years	84.6	3.0	80-89
Education, years	8.8	2.2	6-15
Monthly income, Euros	1014.7	410.5	300-3250

*SD* Standard Deviation.

*MMSE* Mini-Mental State Examination, four item version.

*GDS* Geriatric Depression Scale.

^a^Four participants were excluded from the mortality analyses. Since they did not provide their social security numbers at baseline, mortality data could not be retrieved, leaving us with N = 393 for the mortality analyses.

Note: Number of environmental barriers assessed with the Housing Enabler instrument [24].

Other background information included age, sex, and self-reports of education (years), and monthly income. Depressive symptoms were assessed with the 15-item Geriatric Depression Scale (GDS) [26]. For assessment of cognitive dysfunction, four questions from the Mini Mental State Examination (MMSE) [27] considered sensitive to cognitive dysfunction [28] were used.

### Statistical analyses

Baseline characteristics were described by using means and standard deviations or percentages. Cox regression models for time-dependent predictors [29] were used to assess the association between the number of environmental barriers in each housing section and mortality. The interaction effect between number of functional limitations and number of environmental barriers in each housing section were not significant (smallest for indoor barriers p = 0.270), and so we decided to assess the confounding effect of functional limitations as a main effect in the analyses.

Number of environmental barriers in each housing section and functional limitations were used as time-dependent covariates (values recorded at four measurement points). In the time interval between measurement points, the covariate value was held fixed to the value measured at the beginning of the interval. This approach gave us the possibility to take into account change in the number of environmental barriers and functional limitations over time. For the other covariates as well as for the environmental barriers generating the most P-E fit problems, baseline information was used.

All analyses were performed separately for the outdoor, entrance and indoor sections of the HE. Two separate Cox regression models were constructed for each housing section to study the number of environmental barriers associated with mortality. The first models were adjusted for age and sex, and the second models were adjusted for age, sex, depressive symptoms, cognitive functions, education in years, monthly income, and number of functional limitations.

In order to study the influence on mortality of the environmental barriers generating the most P-E fit problems, proportional hazard Cox regression analyses were performed. Analyses were performed separately for the outdoor, entrance and indoor sections. Five environmental barriers generating the most P-E fit problems in each housing section were included in the models simultaneously and first adjusted for age and sex. The second models were adjusted for age, sex, depressive symptoms, cognitive functions, education in years, monthly income, and number of functional limitations. For the environmental barriers generating the most P-E fit problems, Bonferroni-corrected P-values were calculated by multiplying the model P-values with the number of explanatory variables (5) in the model.

Results are reported as Risk Ratios (RRs) and 95% Confidence Intervals (CI). When the 95% CIs did not include one, or *P* < .05, the differences were regarded as statistically significant. Predictive Analytics SoftWare (PASW) version 18.0 (SPSS Inc., Chicago, IL) was used for the baseline descriptive and proportional hazard Cox regression analyses to analyse the association between environmental barriers generating the most P-E fit problems and mortality. The survival package (version 2.36-5) in the R programming environment (version 2.12.2) was used for the time-dependent Cox regression analyses of the association between the number of environmental barriers in each housing section and mortality [30].

## Results

The mean age of the participants was 84.6 ± 3.02 years; 75% were women. Most of them lived in multi-dwelling blocks in an urban area. At baseline, almost half of the participants needed mobility devices. Further baseline sample characteristics are shown in Table 1. For each housing section, the five most prevalent environmental barriers and the mean number of barriers are shown in Table 2. In each housing section, there were approximately 12 barriers in the outdoor and entrance environments, and almost 39 barriers in the indoor environment.

**Table 2:** The most prevalent environmental barriers in the housing sections, and number of barriers at baseline (n = 397)

**Environmental barrier**			**Occurrence of barriers %**
Outdoors			
Insufficient maneuvering space by the mail box or refuse bin			49.0
Poorly drained paths and roadways			42.9
Unstable walking surface			41.9
No tactile cues of abrupt level changes			41.2
No/too few seating places			37.9
Entrance			
Stairs the only route			46.7
Doors that cannot be fastened in the open position			45.7
Narrow door			45.7
No level area in front of entrance door			42.2
No handrails			39.1
Indoors			
Inappropriate design of door to laundry room			49.7
Toilet with standard height			49.2
Complex maneuvers are needed to use the apparatus			49.2
Apparatus/controls in very high/ inaccessible place in general			48.7
Bathtub			48.5
**Number of environmental barriers**	**Mean**	**SD**	**Range**
Outdoor section (0–33)	12.5	3.5	3-22
Entrance section (0–49)	12.3	5.8	2-32
Indoor section (0–100)	37.9	6.7	18-59

Note: Environmental barriers assessed with the Housing Enabler instrument [24].

A total of 264 (67%) participants died during follow-up. The number of environmental barriers in the outdoor or entrance sections was not associated with mortality when adjusted for age and sex. A higher number of environmental barriers indoors showed a slightly protective effect on mortality (age- and sex-adjusted RR 0.97; 95% CI 0.95-0.99). Further adjustments for number of functional limitations, education in years, monthly income, cognitive functioning and depression had no effect on the results (Table 3).

**Table 3:** The association between number of environmental barriers and mortality (n = 393)

	**Model 1**	**Model 2**
**Environmental section (no. of barriers)**	**RR (95% CI)**	**RR (95% CI)**
Outdoor	0.97 (0.93-1.02)	1.00 (0.96-1.04)
Entrance	0.99 (0.96-1.01)	0.99 (0.97-1.02)
Indoor	0.97 (0.95-0.99)	0.98 (0.96-1.00)

Model 1: Adjusted for age and sex.

Model 2: Adjusted for age, sex, depressive symptoms, cognitive functioning, years of education, monthly income and number of functional limitations.

*RR* Risk Ratio.

*CI* Confidence Interval.

Note: Environmental barriers assessed with the Housing Enabler instrument [24].

Among the environmental barriers that generated the most P-E fit problems in the study sample (Table 4), after full adjustment and Bonferroni correction lack of handrails on stairways was the only environmental barrier associated with higher mortality risk (Bonferroni-corrected p-value = 0.025). The remaining items, especially outdoors, mainly showed a non-significant trend towards increased risk for mortality.

**Table 4:** The association between environmental barriers generating the most Person-Environment fit problems and mortality (n = 393)

	**Model 1**		**Model 2**	
**Environmental barrier**^**a**^	**RR**	**95% CI**	**RR**	**95% CI**
Outdoors				
Path surfaces not level	1.00	0.66-1.52	1.11	0.68-1.81
Refuse room/refuse bin can only be reached via steps	1.42	0.87-2.34	1.73	0.98-3.07
High curbs	1.21	0.91-1.61	1.13	0.79-1.60
No/too few seating places	1.15	0.89-1.50	1.22	0.90-1.67
Inadequate shelter from weather in passenger unloading zone	0.92	0.66-1.28	0.74	0.51-1.08
Entrance				
High threshold/level difference/step	0.84	0.59-1.19	0.93	0.61-1.42
High thresholds and/or steps at the entrance	0.97	0.71-1.32	1.04	0.72-1.49
Doors that cannot be fastened in open position	0.95	0.74-1.22	0.81	0.60-1.09
Stairs the only route	0.88	0.68-1.15	0.88	0.65-1.20
No handrails	1.45	1.12-1.88*	1.55	1.14-2.10*
Indoors				
Wall-mounted cupboards and shelves placed extremely high	1.23	0.94-1.61	1.44	1.03-2.00
Shelves too deep	1.23	0.87-1.75	1.11	0.73-1.66
Storage areas can only be reached via stairs/threshold^b^	1.42	0.86-2.34	1.84	0.95-3.57
No grab bars at shower/bath and/or toilet	0.94	0.74-1.21	0.98	0.73-1.31
Laundry room can only be reached via stairs/threshold^b^	0.72	0.52-1.01	0.68	0.46-1.01

Model 1: All the environmental barriers in each housing section are included in the model simultaneously and adjusted for age and sex.

Model 2: All the environmental barriers in each housing section are included in the model simultaneously and adjusted for age, sex, depressive symptoms, cognitive functioning, years of education, monthly income and number of functional limitations.

^a^Identified by means of the weighted environmental barriers function of the Housing Enabler instrument [19,24], in each housing section listed in falling order, starting with the barrier generating the most person-environment fit problems.

^b^Depending on different housing types, this barrier might be located indoors or outdoors.

*Statistically significant after Bonferroni correction.

When examining the confounders, each additional functional limitation increased mortality risk by almost 20% (RR 1.18; 95% CI 1.10-1.26) and each additional year of age by 10% (RR 1.10; 95% CI 1.05-1.15), while good cognitive capacity (RR 0.51; 95% CI 0.28-0.92) was associated with lower mortality risk. Socioeconomic indicators (income and education) and depressive symptoms were not associated with mortality.

## Discussion

The results of the present study showed that the number of objectively measured environmental barriers in the entrance and immediate outdoor areas of home do not predict mortality among very old, single-living people in Sweden. Unexpectedly, the results indicate that a high number of environmental barriers indoors have a slightly protective effect against mortality risk, even after adjustment for functional limitations. Utilizing the HE weighted environmental barrier approach to examine the environmental barriers that generate the most P-E fit problems in the sample, we found that lack of handrails in entrances was associated with higher mortality risk. To the best of our knowledge, this is the first scientific study of its kind, but given the intriguing result regarding environmental barriers indoors, additional methodological considerations and further research is certainly needed.

Although it is unlikely that there is a direct causal relationship between environmental barriers and mortality, the existence of environmental barriers could influence mobility, participation and overall health in old age, and thus eventually indirectly lead to increased mortality. The association between objectively assessed environmental barriers and mortality has not been widely studied, even though the environmental implications for healthy aging have been acknowledged in several studies. In these studies healthy aging outcomes have been mobility- and disability-related [1], or have targeted general wellbeing and quality of life [31]. When investigating environmental barriers, most studies have used self-reports [5]. It should also be noted that most of the previous studies on the association between environmental features and mortality have focused on environmental features reflecting socioeconomic differences or deprivation. For example, people living in smaller dwellings have increased risk for mortality, compared to those living in larger dwellings, which is partially explained by socio-economic differences [32]. Other studies that have found an association between poor housing conditions and mortality risk [8,33] have also suggested that poor housing conditions reflect poor socio-economic status [33], which in turn increases mortality risk [34]. In our study, socio-economic status did not correlate with mortality risk and had no effect on the association between environmental barriers and mortality. It should be noted that in our study, the socio-economic differences between individuals were small and the number and the variance of environmental barriers was rather low, indicating small differences in housing conditions among the participants and thus probably underestimation of the results. It is also likely that individual-related factors, such as illnesses, become more important than environmental features in predicting mortality risk when people reach very old age. This was demonstrated by the fact that the number of functional limitations was significantly associated with mortality while the number of environmental barriers was not.

In order to operationalize the notion of P-E fit, we chose to use the five environmental barriers in each housing section that, by means of the special weighted barrier function of the HE [19], were identified as those generating the most P-E fit problems in the study sample. In this way, we found that lack of handrails at entrances increased mortality risk. Lack of handrails in the entrance area may cause falls, and falls and fall-related injuries are well-known mortality risks among old and very old people [35]. This environmental barrier may also diminish older people’s possibilities to access outdoor environments, thus restricting their participation in out-of-home activities, in turn leading to physical inactivity [36], increased mobility limitations [5] and constricted life-space of older people, all of which have been found to have an indirect effect on mortality [37,38]. To study the effects of activity restrictions or life-space mobility on mortality is beyond the scope of the present study, but is an important issue for future research. It should also be noted that prior to the Bonferroni correction, the barrier ‘wall-mounted cupboards/shelves placed extremely high in the kitchen’ also emerged as statistically significant. As reaching high up or standing on a chair to take something from an upper shelf, potentially leading to loss of balance and falling, may increase risk for falls and injuries, it is not unreasonable to assume that this particular environmental barrier is associated with mortality. Falling or dropping from a low altitude, such as from a chair, is the most common reason of accidental death among older people [39].

There might be several reasons behind the unexpected finding that a higher number of indoor environmental barriers showed a slightly protective effect on mortality. The most likely explanation would have been health differences, and we did adjust the models for several meaningful health differences. However, it should be noted that since we did not adjust for the influence of diagnoses highly associated with mortality, the residual confounding from conditions such as cancer, cardiovascular diseases, diabetes, etc., is an important explanation. It might also be that the variable used is too coarse, since the mere number of environmental barriers is not at all related to the functional capacity of the individual. Also, given the large total number of items for the indoor section of the HE, the actual composition of barriers making up the sum score for each case varies substantially within the sample. However, we should not rule out the possibility that environmental barriers may maintain the functioning and health of older people by providing physical exercise as integral part of their daily activities, but since few of the indoor environmental barriers assessed by means of the HE are likely to present challenges of that type, we would prefer to refrain from drawing such a conclusion solely on the basis of this study. Nevertheless, the ecological model of ageing indicates that misfit between the environment and the individual (P-E fit) can also occur in situations where the environmental demands are inadequate relative to the person’s capabilities [16]. The threshold at which environmental facilitators turn into barriers and vice versa would be an interesting target for future studies.

Well aware of the limitations of using the number of environmental barriers variable, in order to make more of the importance of the type of environmental barriers rather than count we also utilized the weighted environmental barrier function available in the HE software. However, it should be noted that since the weighted barrier analysis is based on the sample-specific prevalence of functional limitations in relationship to the occurrence of environmental barriers, the barrier item-specific P-E fit score is not specific to the individual’s particular functional status and abilities. That is, some environmental barriers important to one person may not be important to others rendering the meaningful interpretation potentially challenging. More specific analyses require additional in-depth descriptive analyses [40], being beyond the scope of the present study.

Another study limitation to be noted when interpreting the results is the fact that the time between the second and third assessment was rather long (5–6 years) and therefore it is possible that environmental changes occurring during that period would not have been taken into account for deaths that occurred just prior to the third assessment. However, since only 43 (three between the second and third assessment) participants relocated to ordinary housing during the follow-up, environmental changes incurred by relocation are not likely to influence the results. Yet another limitation is that the most of the participants lived in urban areas in Sweden, and thus the environmental barriers, and corresponding health consequences, may be different in rural areas or in other countries. Previous studies have found differences between urban- and rural-dwelling people in physical activity, socio-economic status and health [41,42], but also that urban-dwelling older people have better survival than their rural counterparts [43]. This requires more research, not the least from a cross-national perspective.

It should also be noted that owing to specific inclusion criteria based on the knowledge that single-living older people represent a segment of the ageing population that is particularly vulnerable to environmental demands [21], only those who lived alone were included in the ENABLE-AGE project. We cannot rule out the possibility that environmental barriers affect mortality differently among those living alone compared to among those who are co-habiting. Those who live alone have to face their environmental challenges alone, while those living with others may overcome such barriers with help from another person, or they can avoid performing challenging tasks and activities by asking someone else to take the responsibility for them. This topic also warrants further study.

The strengths of the study are the long follow-up period with several measurement points and the opportunity to use time-dependent analyses on a topic that has not been widely studied. This approach gave us the possibility to take into account changes in the housing environment as well as in the functional capacity of the individual. In addition, environmental features were assessed objectively with an instrument with documented reliability and validity [23].

## Conclusion

Specific environmental problems may increase mortality risk among very-old single-living people. However, the association may be confounded by individuals’ health status which is difficult to fully control for. There is a need for further research on the dynamics of P-E fit during the ageing process. As people become older they spend more and more of their time at home or in its immediate surroundings, and hence there is a need to better understand the health consequences of the everyday living environment.

## Abbreviations

ENABLE-AGE project: Enabling Autonomy, Participation, and Well-Being in Old Age: The Home Environment as a Determinant for Healthy Ageing project; GDS: Geriatric Depression Scale; HE: Housing Enabler; MMSE: Mini Mental State Examination; P-E fit: Person-Environment fit.

## Competing interests

S. Iwarsson, (together with B. Slaug) is the copyright holder and owner of the Housing Enabler (HE) methodology, provided as a commercial product (see http://www.enabler.nu). The remaining authors declare no competing interests.

## Authors’ contributions

The authors are justifiably credited with authorship, according to the authorship criteria. Rantakokko had full access to all the data gathered for the study and takes full responsibility for the integrity of the data and the accuracy of the data analyses. In detail, MR: conception, design, analysis and interpretation of the data, writing the article; TT: Statistical expertise, conception, design, interpretation of the data, critical revision of the article; TR: conception, design, critical revision of the article; MH: data collection, critical revision of the article; SI: conception, design, data collection, obtaining funding, critical revision of the article. SI was also the PI of the original ENABLE-AGE project. All the authors approved the final manuscript.

## Pre-publication history

The pre-publication history for this paper can be accessed here:

http://www.biomedcentral.com/1471-2458/13/783/prepub
